# 2-(4-Methyl­phen­yl)-7-(2-methyl­prop­oxy)-4*H*-chromen-4-one–6-chloro-2-(4-methyl­phen­yl)-7-(2-methyl­prop­oxy)-4*H*-chromen-4-one (19/1)

**DOI:** 10.1107/S1600536812033430

**Published:** 2012-07-28

**Authors:** Vijay M. Barot, Mukesh M. Jotani, Jeshal G. Maheta

**Affiliations:** a Department of Chemistry, Smt. S. M. Panchal Science College, Talod, Gujarat 383 215, India; b Department of Physics, Bhavan’s Sheth R. A. College of Science, Ahmedabad, Gujarat 380 001, India

## Abstract

The title co-crystal, 0.95C_20_H_20_O_3_·0.05C_20_H_19_ClO_3_, arises as the chloride carried over during the synthesis shares a position with an aromatic H atom; the partial occupancies are 0.947 (2) and 0.053 (2) for H and Cl, respectively. The mol­ecular structure is stabilized by intra­molecular C—H⋯O contacts, forming pseudo five- and six-membered rings with *S*(5) and *S*(6) graph-set motifs, respectively. The crystal structure features π–π stacking inter­actions between the centroids of the central fused ring systems [centroid–centroid distance = 3.501 (2) Å].

## Related literature

For background to flavones, see: Hollman *et al.* (1997 ▶); Yao *et al.* (2004 ▶). For the biological activity of flavones, see: Harborne & Williams (2000 ▶); Khan & Hasan (2003 ▶); Qin *et al.* (2008 ▶); Mota *et al.* (2009 ▶); Prakash *et al.* (2009 ▶). For hydrogen-bond motifs, see: Bernstein *et al.* (1995 ▶).
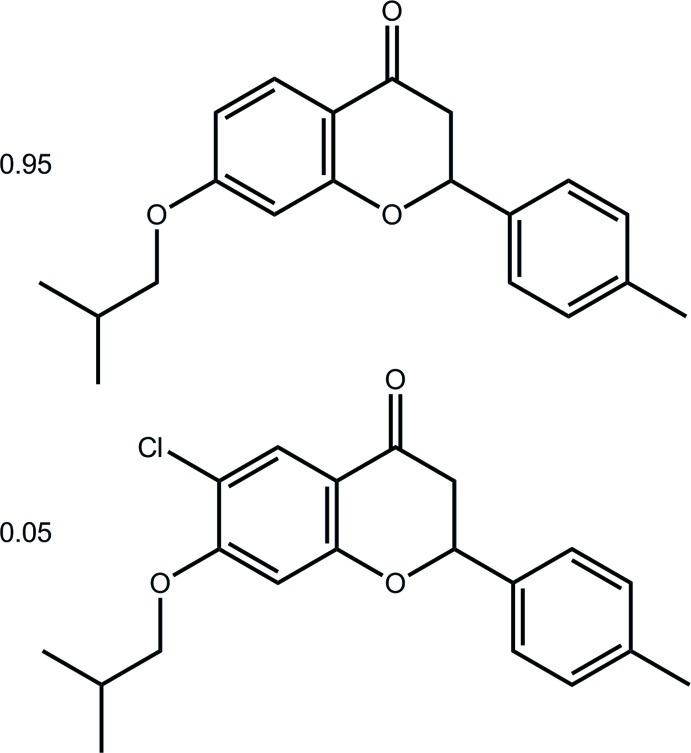


## Experimental

### 

#### Crystal data

0.95C_20_H_20_O_3_·0.05C_20_H_19_ClO_3_
*M* *_r_* = 310.19
Triclinic, 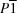

*a* = 9.0371 (2) Å
*b* = 9.6216 (2) Å
*c* = 11.0308 (3) Å
α = 75.171 (2)°
β = 65.865 (2)°
γ = 69.833 (1)°
*V* = 814.20 (4) Å^3^
*Z* = 2
Mo *K*α radiation
μ = 0.09 mm^−1^
*T* = 293 K
0.30 × 0.20 × 0.20 mm

#### Data collection

Bruker Kappa APEXII CCD diffractometer
Absorption correction: multi-scan (*SADABS*; Bruker, 2004 ▶) *T*_min_ = 0.973, *T*_max_ = 0.982
18174 measured reflections
3847 independent reflections
2737 reflections with *I* > 2σ(*I*)
*R*_int_ = 0.026

#### Refinement

*R*[*F* ^2^ > 2σ(*F* ^2^)] = 0.048
*wR*(*F* ^2^) = 0.136
*S* = 1.06
3847 reflections
219 parameters
H-atom parameters constrained
Δρ_max_ = 0.34 e Å^−3^
Δρ_min_ = −0.17 e Å^−3^

### 

Data collection: *APEX2* (Bruker, 2004 ▶); cell refinement: *APEX2* and *SAINT* (Bruker, 2004 ▶); data reduction: *SAINT* and *XPREP* (Bruker, 2004 ▶); program(s) used to solve structure: *SIR92* (Altomare *et al.*, 1999 ▶); program(s) used to refine structure: *SHELXL97* (Sheldrick, 2008 ▶); molecular graphics: *PLATON* (Spek, 2009 ▶); software used to prepare material for publication: *SHELXL97*.

## Supplementary Material

Crystal structure: contains datablock(s) global, I. DOI: 10.1107/S1600536812033430/tk5116sup1.cif

Structure factors: contains datablock(s) I. DOI: 10.1107/S1600536812033430/tk5116Isup2.hkl

Supplementary material file. DOI: 10.1107/S1600536812033430/tk5116Isup3.cml

Additional supplementary materials:  crystallographic information; 3D view; checkCIF report

## Figures and Tables

**Table 1:** Hydrogen-bond geometry (Å, °)

*D*—H⋯*A*	*D*—H	H⋯*A*	*D*⋯*A*	*D*—H⋯*A*
C19—H19⋯O3	0.93	2.38	2.702 (2)	100
C1—H1*A*⋯O1	0.96	2.58	2.900 (2)	100

**Fig. 1.**
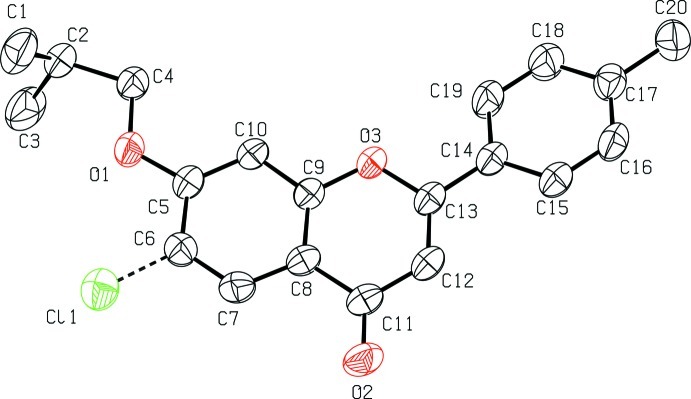
Molecular structure of (I) showing 50% probability displacement ellipsoids. The hydrogen atoms are omitted for clarity.

**Fig. 2.**
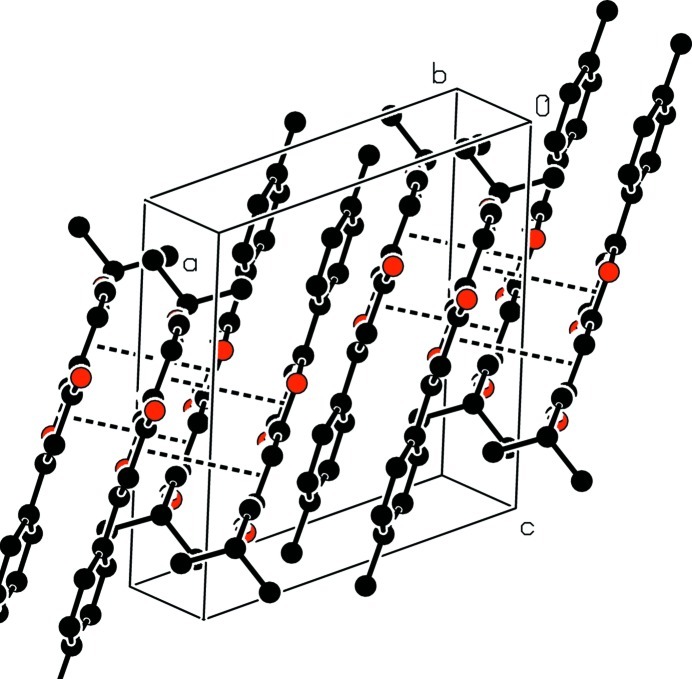
Crystal packing showing π—π stacking interactions indicated by dashed lines. H atoms are omitted for clarity.

## supplementary crystallographic information

### Comment

Flavones can be considered as the derivatives of a parent compound 2-phenylchromen containing varying degrees of hydroxylation and methoxylation (Yao *et al.*, 2004). Also, flavones and their derivatives at different oxidation level are well known naturally occurring oxygen-containing potent anti-oxidant heterocyclic compounds as they chelate ions, scavenge oxygen free radicals and prevent the oxidation of low density lipoprotein (Hollman *et al.*, 1997). Both natural and synthetic flavones possess a wide spectrum of biological activities such as anti-bacterial, anti-fungal, anti-inflammatory, anti-cancer, *etc*. (Prakash *et al.*, 2009; Mota *et al.*, 2009; Qin *et al.*, 2008; Khan & Hasan, 2003). The continuous search for the synthesis of new derivatives in this group due to their medicinal importance (Harborne & Williams, 2000) is the main motivation for the study of title flavone molecule. In view of their importance, the title compound, 2-(4-methylphenyl)-7-(2-methylpropoxy)-4*H*-chromen-4-one (I) was synthesized and its crystal structure studied.

The molecular structure of (I), Fig. 1, consists of a central chromen ring extended by a toluene ring on one side and a propoxy moiety on other side. The bicyclic chromen ring is almost coplanar with C8, C12 and C13 atoms have maximum respective deviations of -0.205 (16), 0.0185 (16) and 0.158 (15) Å with respect to least square plane through it. The fractional chlorine atom remains in the molecule of (I) during the synthesis and its presence is confirmed during the structural refinement as it shares a position with the aromatic hydrogen H19 atom; the partial occupancies are 0.947 (2) and 0.053 (2) for H19 and Cl1 atoms, respectively. In the absence of hydrogen bonds, the crystal structure of (I) is stabilized by intramolecular short C—H···O contacts forming pseudo five- and six-membered rings of *S*(5) and *S*(6) graph-set motif (Bernstein *et al.*, 1995), Table 1, and by π—π stacking interactions between symmetry related fused chromen rings (*Cg*1—*Cg*2 (2 - *x*, -*y*, 1 - *z*) = 3.501 (2) Å; *Cg*1 = C5—C10 and *Cg*2 = O3/C9/C8/C11—C13), Fig. 2.

### Experimental

(2*E*)-1-[2-Hydroxy-4-(2-methylpropoxy)phenyl]-3-(4-methylphenyl)prop-2-ene-1-one (0.01 mol) was dissolved in DMSO (30 ml) and iodine, in crystalline powder form, was added. The mixture was then heated at about 140–145 °C for 1 h and the reaction was monitored by continuous TLC. The resulting solution was cooled and diluted with water after the completion of reaction. Excess iodine was removed by filtering and washing the product with 20% aqueous sodium bisulphite. The crude product was then purified by column chromatography using toluene-ethyl acetate (10:1) as the mobile phase and a silica gel as the stationary phase. The melting point was measured on an Electro thermal 9200 apparatus and is uncorrected (Yield: 68%, *M*.pt: 442 K). The colourless block-shaped crystals of the title compound were obtained by re-crystallization from its ethanol solution.

### Refinement

Carbon-bound H-atoms were placed in calculated positions (C—H = 0.93 to 0.98 Å) and were included in the refinement in the riding model approximation with *U*_iso_(H) set to 1.2–1.5*U*_eq_(C). A reflection affected by the beam stop, *i.e*. (0 0 1), was omitted from the final refinement.

### Crystal data

**Table:**

0.95C_20_H_20_O_3_·0.05C_20_H_19_ClO_3_	*Z* = 2
*M**_r_* = 310.19	*F*(000) = 329.6
Triclinic, *P*1	*D*_x_ = 1.265 Mg m^−^^3^
Hall symbol: -P 1	Mo *K*α radiation, λ = 0.71069 Å
*a* = 9.0371 (2) Å	Cell parameters from 5339 reflections
*b* = 9.6216 (2) Å	θ = 3.0–25.0°
*c* = 11.0308 (3) Å	µ = 0.09 mm^−^^1^
α = 75.171 (2)°	*T* = 293 K
β = 65.865 (2)°	Block, colourless
γ = 69.833 (1)°	0.30 × 0.20 × 0.20 mm
*V* = 814.20 (4) Å^3^	

### Data collection

**Table:**

Bruker Kappa APEXII CCD diffractometer	3847 independent reflections
Radiation source: fine-focus sealed tube	2737 reflections with *I* > 2σ(*I*)
Graphite monochromator	*R*_int_ = 0.026
ω and φ scan	θ_max_ = 27.8°, θ_min_ = 2.3°
Absorption correction: multi-scan (*SADABS*; Bruker, 2004)	*h* = −11→11
*T*_min_ = 0.973, *T*_max_ = 0.982	*k* = −12→12
18174 measured reflections	*l* = −14→14

### Refinement

**Table:**

Refinement on *F*^2^	Primary atom site location: structure-invariant direct methods
Least-squares matrix: full	Secondary atom site location: difference Fourier map
*R*[*F*^2^ > 2σ(*F*^2^)] = 0.048	Hydrogen site location: inferred from neighbouring sites
*wR*(*F*^2^) = 0.136	H-atom parameters constrained
*S* = 1.06	*w* = 1/[σ^2^(*F*_o_^2^) + (0.0582*P*)^2^ + 0.1669*P*] where *P* = (*F*_o_^2^ + 2*F*_c_^2^)/3
3847 reflections	(Δ/σ)_max_ = 0.005
219 parameters	Δρ_max_ = 0.34 e Å^−^^3^
0 restraints	Δρ_min_ = −0.17 e Å^−^^3^

### Special details

**Table:**

Geometry. All e.s.d.'s (except the e.s.d. in the dihedral angle between two l.s. planes) are estimated using the full covariance matrix. The cell e.s.d.'s are taken into account individually in the estimation of e.s.d.'s in distances, angles and torsion angles; correlations between e.s.d.'s in cell parameters are only used when they are defined by crystal symmetry. An approximate (isotropic) treatment of cell e.s.d.'s is used for estimating e.s.d.'s involving l.s. planes.
Refinement. Refinement of *F*^2^ against ALL reflections. The weighted *R*-factor *wR* and goodness of fit *S* are based on *F*^2^, conventional *R*-factors *R* are based on *F*, with *F* set to zero for negative *F*^2^. The threshold expression of *F*^2^ > σ(*F*^2^) is used only for calculating *R*-factors(gt) *etc*. and is not relevant to the choice of reflections for refinement. *R*-factors based on *F*^2^ are statistically about twice as large as those based on *F*, and *R*- factors based on ALL data will be even larger.

### Fractional atomic coordinates and isotropic or equivalent isotropic displacement parameters (Å^2^)

**Table:**

	*x*	*y*	*z*	*U*_iso_*/*U*_eq_	Occ. (<1)
O1	0.96616 (14)	−0.37259 (11)	0.75225 (11)	0.0496 (3)	
O2	0.73241 (16)	0.33677 (12)	0.64615 (12)	0.0611 (3)	
O3	0.72302 (13)	0.01688 (10)	0.47020 (10)	0.0427 (3)	
C1	0.9533 (3)	−0.6722 (2)	0.8857 (2)	0.0753 (6)	
H1B	1.0079	−0.7685	0.9223	0.113*	
H1C	0.8465	−0.6739	0.8881	0.113*	
H1A	0.9360	−0.5981	0.9377	0.113*	
C2	1.0620 (2)	−0.63483 (17)	0.74312 (17)	0.0502 (4)	
H2	1.0725	−0.7099	0.6924	0.060*	
C3	1.2383 (2)	−0.6446 (2)	0.7309 (3)	0.0807 (7)	
H3C	1.3039	−0.6216	0.6384	0.121*	
H3A	1.2900	−0.7440	0.7645	0.121*	
H3B	1.2327	−0.5746	0.7820	0.121*	
C4	0.9825 (2)	−0.48407 (16)	0.67936 (16)	0.0464 (4)	
H4A	1.0526	−0.4655	0.5862	0.056*	
H4B	0.8726	−0.4815	0.6828	0.056*	
C5	0.90580 (18)	−0.22724 (16)	0.70937 (15)	0.0412 (3)	
C6	0.9127 (2)	−0.12487 (17)	0.77583 (16)	0.0471 (4)	
H6	0.9567	−0.1590	0.8443	0.057*	0.9470 (18)
C7	0.8550 (2)	0.02482 (17)	0.74021 (16)	0.0467 (4)	
H7	0.8601	0.0919	0.7851	0.056*	
C8	0.78841 (17)	0.07948 (16)	0.63740 (14)	0.0398 (3)	
C9	0.78410 (17)	−0.02488 (15)	0.57358 (14)	0.0373 (3)	
C10	0.84152 (17)	−0.17760 (15)	0.60726 (14)	0.0397 (3)	
H10	0.8369	−0.2447	0.5623	0.048*	
C11	0.72965 (19)	0.23849 (16)	0.59441 (15)	0.0446 (4)	
C12	0.67062 (19)	0.27121 (16)	0.48452 (16)	0.0455 (4)	
H12	0.6314	0.3707	0.4520	0.055*	
C13	0.66968 (17)	0.16449 (15)	0.42691 (15)	0.0406 (3)	
C14	0.61702 (18)	0.18806 (16)	0.31181 (15)	0.0425 (3)	
C15	0.5271 (2)	0.32770 (17)	0.26928 (16)	0.0485 (4)	
H15	0.4986	0.4081	0.3142	0.058*	
C16	0.4797 (2)	0.34794 (17)	0.16082 (17)	0.0507 (4)	
H16	0.4203	0.4427	0.1334	0.061*	
C17	0.5174 (2)	0.23220 (18)	0.09144 (16)	0.0489 (4)	
C18	0.6088 (3)	0.09398 (19)	0.13338 (19)	0.0627 (5)	
H18	0.6381	0.0141	0.0877	0.075*	
C19	0.6579 (2)	0.07170 (18)	0.24164 (19)	0.0587 (5)	
H19	0.7191	−0.0227	0.2678	0.070*	
C20	0.4595 (2)	0.2551 (2)	−0.02344 (18)	0.0628 (5)	
H20A	0.5374	0.2937	−0.1039	0.094*	
H20B	0.3498	0.3249	−0.0052	0.094*	
H20C	0.4543	0.1615	−0.0348	0.094*	
Cl1	0.9913 (10)	−0.1791 (10)	0.9090 (9)	0.060 (3)	0.0530 (18)

### Atomic displacement parameters (Å^2^)

**Table:**

	*U*^11^	*U*^22^	*U*^33^	*U*^12^	*U*^13^	*U*^23^
O1	0.0659 (7)	0.0342 (6)	0.0515 (7)	−0.0061 (5)	−0.0312 (6)	−0.0029 (5)
O2	0.0839 (9)	0.0380 (6)	0.0665 (8)	−0.0159 (6)	−0.0280 (7)	−0.0138 (5)
O3	0.0506 (6)	0.0282 (5)	0.0497 (6)	−0.0065 (4)	−0.0231 (5)	−0.0029 (4)
C1	0.1004 (16)	0.0513 (11)	0.0633 (12)	−0.0210 (11)	−0.0291 (12)	0.0109 (9)
C2	0.0599 (10)	0.0339 (8)	0.0580 (10)	−0.0101 (7)	−0.0277 (8)	−0.0004 (7)
C3	0.0635 (12)	0.0462 (10)	0.132 (2)	−0.0063 (9)	−0.0503 (13)	0.0042 (11)
C4	0.0549 (9)	0.0370 (8)	0.0483 (9)	−0.0088 (7)	−0.0225 (7)	−0.0050 (7)
C5	0.0415 (8)	0.0339 (7)	0.0433 (8)	−0.0072 (6)	−0.0135 (7)	−0.0037 (6)
C6	0.0529 (9)	0.0455 (9)	0.0457 (9)	−0.0121 (7)	−0.0212 (7)	−0.0062 (7)
C7	0.0517 (9)	0.0428 (9)	0.0487 (9)	−0.0137 (7)	−0.0164 (7)	−0.0121 (7)
C8	0.0381 (7)	0.0348 (7)	0.0422 (8)	−0.0108 (6)	−0.0075 (6)	−0.0079 (6)
C9	0.0356 (7)	0.0344 (7)	0.0382 (8)	−0.0092 (6)	−0.0102 (6)	−0.0039 (6)
C10	0.0424 (8)	0.0325 (7)	0.0428 (8)	−0.0091 (6)	−0.0142 (7)	−0.0055 (6)
C11	0.0455 (8)	0.0356 (8)	0.0477 (9)	−0.0117 (6)	−0.0086 (7)	−0.0099 (6)
C12	0.0479 (9)	0.0287 (7)	0.0521 (9)	−0.0057 (6)	−0.0146 (7)	−0.0045 (6)
C13	0.0376 (7)	0.0305 (7)	0.0459 (8)	−0.0066 (6)	−0.0111 (6)	−0.0021 (6)
C14	0.0424 (8)	0.0321 (7)	0.0478 (9)	−0.0083 (6)	−0.0149 (7)	−0.0010 (6)
C15	0.0510 (9)	0.0331 (8)	0.0560 (10)	−0.0065 (7)	−0.0190 (8)	−0.0040 (7)
C16	0.0494 (9)	0.0361 (8)	0.0589 (10)	−0.0077 (7)	−0.0224 (8)	0.0062 (7)
C17	0.0473 (9)	0.0475 (9)	0.0483 (9)	−0.0161 (7)	−0.0169 (7)	0.0039 (7)
C18	0.0868 (13)	0.0410 (9)	0.0661 (12)	−0.0078 (9)	−0.0392 (11)	−0.0094 (8)
C19	0.0780 (12)	0.0320 (8)	0.0685 (11)	0.0000 (8)	−0.0408 (10)	−0.0060 (7)
C20	0.0691 (12)	0.0642 (12)	0.0582 (11)	−0.0207 (9)	−0.0303 (9)	0.0033 (9)
Cl1	0.053 (5)	0.061 (6)	0.059 (5)	−0.016 (4)	−0.016 (4)	−0.001 (4)

### Geometric parameters (Å, º)

**Table:**

O1—C5	1.3503 (17)	C7—H7	0.9300
O1—C4	1.4333 (17)	C8—C9	1.3844 (19)
O2—C11	1.2348 (17)	C8—C11	1.457 (2)
O3—C13	1.3589 (16)	C9—C10	1.3852 (19)
O3—C9	1.3762 (17)	C10—H10	0.9300
C1—C2	1.503 (3)	C11—C12	1.439 (2)
C1—H1B	0.9600	C12—C13	1.343 (2)
C1—H1C	0.9600	C12—H12	0.9300
C1—H1A	0.9600	C13—C14	1.470 (2)
C2—C4	1.513 (2)	C14—C19	1.383 (2)
C2—C3	1.514 (2)	C14—C15	1.386 (2)
C2—H2	0.9800	C15—C16	1.377 (2)
C3—H3C	0.9600	C15—H15	0.9300
C3—H3A	0.9600	C16—C17	1.380 (2)
C3—H3B	0.9600	C16—H16	0.9300
C4—H4A	0.9700	C17—C18	1.380 (2)
C4—H4B	0.9700	C17—C20	1.499 (2)
C5—C10	1.379 (2)	C18—C19	1.380 (2)
C5—C6	1.401 (2)	C18—H18	0.9300
C6—C7	1.364 (2)	C19—H19	0.9300
C6—Cl1	1.772 (9)	C20—H20A	0.9600
C6—H6	0.9300	C20—H20B	0.9600
C7—C8	1.400 (2)	C20—H20C	0.9600
			
C5—O1—C4	118.87 (11)	O3—C9—C8	121.81 (12)
C13—O3—C9	119.23 (11)	O3—C9—C10	115.07 (12)
C2—C1—H1B	109.5	C8—C9—C10	123.12 (13)
C2—C1—H1C	109.5	C5—C10—C9	118.11 (13)
H1B—C1—H1C	109.5	C5—C10—H10	120.9
C2—C1—H1A	109.5	C9—C10—H10	120.9
H1B—C1—H1A	109.5	O2—C11—C12	122.81 (14)
H1C—C1—H1A	109.5	O2—C11—C8	123.12 (14)
C1—C2—C4	112.08 (15)	C12—C11—C8	114.06 (12)
C1—C2—C3	112.10 (17)	C13—C12—C11	122.91 (13)
C4—C2—C3	110.95 (14)	C13—C12—H12	118.5
C1—C2—H2	107.1	C11—C12—H12	118.5
C4—C2—H2	107.1	C12—C13—O3	121.86 (13)
C3—C2—H2	107.1	C12—C13—C14	126.37 (13)
C2—C3—H3C	109.5	O3—C13—C14	111.77 (12)
C2—C3—H3A	109.5	C19—C14—C15	118.04 (14)
H3C—C3—H3A	109.5	C19—C14—C13	120.87 (13)
C2—C3—H3B	109.5	C15—C14—C13	121.09 (14)
H3C—C3—H3B	109.5	C16—C15—C14	120.31 (14)
H3A—C3—H3B	109.5	C16—C15—H15	119.8
O1—C4—C2	107.95 (12)	C14—C15—H15	119.8
O1—C4—H4A	110.1	C15—C16—C17	122.14 (14)
C2—C4—H4A	110.1	C15—C16—H16	118.9
O1—C4—H4B	110.1	C17—C16—H16	118.9
C2—C4—H4B	110.1	C16—C17—C18	117.17 (15)
H4A—C4—H4B	108.4	C16—C17—C20	121.40 (15)
O1—C5—C10	124.33 (13)	C18—C17—C20	121.43 (16)
O1—C5—C6	115.33 (13)	C19—C18—C17	121.49 (16)
C10—C5—C6	120.34 (13)	C19—C18—H18	119.3
C7—C6—C5	120.05 (14)	C17—C18—H18	119.3
C7—C6—Cl1	116.7 (3)	C18—C19—C14	120.84 (15)
C5—C6—Cl1	123.2 (3)	C18—C19—H19	119.6
C7—C6—H6	120.0	C14—C19—H19	119.6
C5—C6—H6	120.0	C17—C20—H20A	109.5
C6—C7—C8	121.26 (13)	C17—C20—H20B	109.5
C6—C7—H7	119.4	H20A—C20—H20B	109.5
C8—C7—H7	119.4	C17—C20—H20C	109.5
C9—C8—C7	117.11 (13)	H20A—C20—H20C	109.5
C9—C8—C11	120.11 (13)	H20B—C20—H20C	109.5
C7—C8—C11	122.75 (13)		
			
C5—O1—C4—C2	176.62 (13)	C7—C8—C11—O2	−0.8 (2)
C1—C2—C4—O1	62.80 (18)	C9—C8—C11—C12	0.0 (2)
C3—C2—C4—O1	−63.37 (19)	C7—C8—C11—C12	178.16 (14)
C4—O1—C5—C10	7.0 (2)	O2—C11—C12—C13	179.05 (15)
C4—O1—C5—C6	−172.61 (13)	C8—C11—C12—C13	0.1 (2)
O1—C5—C6—C7	179.97 (14)	C11—C12—C13—O3	0.9 (2)
C10—C5—C6—C7	0.3 (2)	C11—C12—C13—C14	−177.83 (14)
O1—C5—C6—Cl1	−1.0 (4)	C9—O3—C13—C12	−1.8 (2)
C10—C5—C6—Cl1	179.3 (3)	C9—O3—C13—C14	177.06 (12)
C5—C6—C7—C8	−0.1 (2)	C12—C13—C14—C19	164.68 (16)
Cl1—C6—C7—C8	−179.2 (3)	O3—C13—C14—C19	−14.1 (2)
C6—C7—C8—C9	−0.1 (2)	C12—C13—C14—C15	−14.5 (2)
C6—C7—C8—C11	−178.39 (14)	O3—C13—C14—C15	166.74 (13)
C13—O3—C9—C8	1.8 (2)	C19—C14—C15—C16	0.4 (2)
C13—O3—C9—C10	−177.59 (12)	C13—C14—C15—C16	179.60 (14)
C7—C8—C9—O3	−179.21 (13)	C14—C15—C16—C17	0.6 (2)
C11—C8—C9—O3	−0.9 (2)	C15—C16—C17—C18	−1.4 (2)
C7—C8—C9—C10	0.2 (2)	C15—C16—C17—C20	177.83 (15)
C11—C8—C9—C10	178.47 (13)	C16—C17—C18—C19	1.2 (3)
O1—C5—C10—C9	−179.90 (13)	C20—C17—C18—C19	−178.04 (17)
C6—C5—C10—C9	−0.3 (2)	C17—C18—C19—C14	−0.2 (3)
O3—C9—C10—C5	179.46 (12)	C15—C14—C19—C18	−0.6 (3)
C8—C9—C10—C5	0.0 (2)	C13—C14—C19—C18	−179.81 (16)
C9—C8—C11—O2	−179.02 (14)		

### Hydrogen-bond geometry (Å, º)

**Table:**

*D*—H···*A*	*D*—H	H···*A*	*D*···*A*	*D*—H···*A*
C19—H19···O3	0.93	2.38	2.702 (2)	100
C1—H1*A*···O1	0.96	2.58	2.900 (2)	100
